# Traditional and non-traditional lipid parameters as risk factors for sudden sensorineural hearing loss

**DOI:** 10.1016/j.bjorl.2024.101435

**Published:** 2024-04-22

**Authors:** Xiaoyan Chen, Zhong Zheng, Ximeng Liu, Jianuo Huang, Daoyu Xie, Yanmei Feng

**Affiliations:** a Shanghai Jiao Tong University School of Medicine affiliated Sixth People's Hospital, Department of Otolaryngology-Head and Neck Surgery, Shanghai, China; b Shanghai Key Laboratory of Sleep Disordered Breathing, Shanghai, China; c Hangzhou Normal University, Zhejiang Province, China; d Affiliated Hospital of Hangzhou Normal University, Department of Otolaryngology-Head and Neck Surgery, Zhejiang Province, China

**Keywords:** Sudden sensorineural hearing loss, Lipids, Biomarker, Risk factor

## Abstract

**Objective:**

The purpose was to explore the effects of traditional and non-traditional lipid parameters on Sudden Sensorineural Hearing Loss (SSNHL).

**Methods:**

The study included 452 patients diagnosed with SSNHL, among whom 206 patients had a level of hearing improvement ≥10 dB after one month of follow-up. A propensity score-matched (2:1) control group was used. Conditional and unconditional logistic regression were used to analyze the risk factors for SSNHL.

**Results:**

Patients with SSNHL had a higher risk of concomitant hypertension and elevated atherosclerogenic lipid levels, with apolipoprotein B and apolipoprotein E identified as independent risk factors for the onset of SSNHL. Additionally, the Lipid Comprehensive Index (LCI) was an independent risk factor for the degree of hearing loss. A positive linear correlation was revealed between triglyceride, non-high-density lipoprotein cholesterol, atherogenic index, Castelli risk index, atherogenic index of plasma, LCI and hearing loss. However, no linear relationship was observed between hearing gain and any lipid parameters. When Total Cholesterol (TC) was in the range of borderline high, the treatment effect was the best. However, the statistical significance disappeared upon adjusting for confounding factors.

**Conclusion:**

Patients with SSNHL exhibited markedly dysregulated lipid metabolism. Elevated serum lipid levels may be a causative factor in auditory impairment and can influence the extent of hearing loss. Promptly improving cochlear microcirculation may benefit patients with borderline elevated TC.

**Level of evidence:**

4.

## Highlights

• SSNHL patients had a higher risk of concomitant hypertension and elevated atherosclerogenic lipid levels.
• APOB, APOE, and LCI were identified as independent risk factors.
• Certain lipids showed a positive linear correlation with hearing loss.
• When TC was in borderline high range, the treatment effect was the best.

## Introduction

Sudden Sensorineural Hearing Loss (SSNHL) is characterized by a sensorineural hearing loss of 30 dB or more at more than three consecutive frequencies within three days.^1^ Its etiology is complex including microcirculatory disturbances, abnormal autoimmunity, viral infections, and more, resulting in a significant economic and psychophysiological burden. The cochlea is an organ characterized by a highly active metabolism with terminal blood vessels lacking collateral circulation, that is susceptible to changes in blood flow, making it prone to ischemia and hypoxia.^2^ The mechanisms by which dyslipidemia may contribute to microcirculatory disorders are as follows: (1) The elevated blood viscosity, leading to impairing oxygen carrying capacity; (2) Activation of the fibrinolytic system, leading to microvascular embolism; (3) The damage to the ultrastructure of stria vascularis or hair cells caused by oxidized lipid; (4) Inhibition of vasorelaxation factors, resulting in persistent constriction of capillaries.^3^, ^4^ A prospective study found that the incidence of SSNHL in the hyperlipidemia cohort was 1.62 times higher than in the non-hyperlipidemia cohort. The adjusted hazard ratio was 1.60 (95% CI 1.39–1.85) after controlling for confounding factors.^5^ In summary, dyslipidemia was considered to be one of the causes of SSNHL.

Mounting evidence has suggested that single lipid parameters may not be the most powerful predictors of atherosclerotic disease development. Many comprehensive lipid indices, such as non-High-Density Lipoprotein Cholesterol (nonHDL-C), Atherogenic Index of Plasma (AIP), Atherogenic Index (AI), the Atherogenic Index (ATH index), Castelli Risk Index-I (CRI-I), Castelli Risk Index-II (CRI-II), Lipid Comprehensive Index (LCI), comprehensive Lipid Tetrad Index (cLTI), and comprehensive Lipid Pentad Index (cLPI), have better predictive efficiency because they represent different lipid components to reflect the lipid metabolism status of the human body.^6^, ^7^, ^8^, ^9^ This study included the AI, LCI, cLTI, and cLPI for the first time and compared the effect of traditional lipid parameters (Total Cholesterol [TC], Triglyceride [TG], Low-Density Lipoprotein Cholesterol [LDL-C)], High-Density Lipoprotein Cholesterol [HDL-C]) and non-traditional lipid parameters (Apolipoprotein A-1 [APOA1], Apolipoprotein-B [APOB], Apolipoprotein E [APOE], lipoprotein a, AIP, AI, ATH index, CRI-I, CRI-II, nonHDL-C, LCI, cLTI, cLPI) on the onset and prognosis of SSNHL.

## Methods

### Study population

From August 2018 to December 2022, the baseline data of 775 patients with SSNHL and physical examination participants who underwent ENT (ear, nose, and throat) examinations and audiological tests without abnormalities during the same period were collected. Exclusion criteria for all participants: (1) Suffering from vestibular migraine, Meniere's disease, conductive hearing loss, ear surgery, structural abnormalities, and other sensorineural hearing loss; (2) Suffering from infectious diseases, diabetes, thyroid diseases, severe organ dysfunction, malignant tumors, autoimmune disease, and liver and kidney dysfunction that affect metabolism were excluded; (3) Incomplete follow-up data; (4) Under 18-years-old; (5) Having a family history and previous history of sensorineural hearing loss; (6) Having binaural or previous SSNHL; (7) Receiving treatment before enrollment; and (8) Being pregnant or nursing. 452 patients with SSNHL were finally included and propensity score matching was used to match the age and gender of the physical examination participants in a 2:1 ratio. The research procedure followed the Helsinki Declaration and was approved by the Ethics Committee. Informed consent was obtained from all participants.

### Data collection

Primary baseline data and disease characteristics were collected. Pure tone audiometry was assessed in a standard masked room from 0.25 to 8 kHz before treatment and one month after treatment. Structural abnormalities and tumors were excluded through imaging examination of the inner ear. Before glucocorticoid treatment, fasting serum was obtained to measure the levels of APOA1 (mg/dL), APOB (mg/dlL), APOE (mg/dL), TC (mmoL/L), TG (mmoL/L), LDL-C (mmoL/L), HDL-C (mmoL/L), and lipoprotein a (mg/dL). Non-HDL-C (TC-HDL-C), AIP (log (TG/HDL-C)), AI (non HDL-C/HDL-C), ATH index[(TC-HDL-C) × (APOB)/(HDL-C × APOA-I)], CRI-I (TC/HDL-C), CRI-II (LDL-C/HDL-C), LCI (TC × TG × LDL/HDL-C), cLTI([TC × TG × lipoproteina]/HDL) and cLPI ((TC × TG × LPA × APOB-100)/APOA1) were calculated.

### Treatment and evaluation

All patients were treated with a 7-day course of systemic glucocorticoid (prednisone 1 mg/kg/day for 3–5 days, maximum dose ≤60 mg/d, followed by a reduced dosage for the remaining days according to the hearing improvement). Retroaural injection involved remedial treatment (dexamethasone 5 mg every other day for 4–5 doses). Batroxobin, neurotrophic factor (mecobalamine) and antioxidant (ginkgo biloba extract) were also used according to Chinese guidelines. The patients with hypertension and hyperlipidemia, antihypertensive and lipid-lowering treatment were adopted.^10^

Following the World Health Organization’s (WHO) first World Hearing Report on 2 March 2021, hearing loss was classified into mild-moderate (< 50 dB) and moderately severe-to-anacusia (≥ 50 dB) by the Pure Tone Average (PTA) at 0.5, 1, 2 and 4 kHz.^11^ The AAO_HNS (American Academy of Otolaryngology-Head and Neck) criteria was used to calculate hearing loss and gain according to the PTA at 0.25–8 kHz, based on which SSNHL patients were divided into the effective group (hearing gain ≥10 dB, or whose hearing was restored to normal or unaffected contralateral hearing thresholds) and ineffective group (hearing gain <10 dB).^1^ According to the impaired frequency, SSNHL were divided into different subtypes: partial frequency descending SSNHL (PF-SSNHL; including low-frequency descending type [≤1 kHz] and high-frequency descending type [≥2 kHz]) and all frequency descending SSNHL (AF-SSNHL; including flat type and total deafness).^10^

### Statistical analysis

Statistical analysis was conducted using the software Statistical Product and Service Solutions 26.0 and R-Studio 4.0.4. Non-normally distributed variables were described by quartile and analyzed using Mann-Whitney *U*-test. Categorical variables were expressed as percentages and analyzed using the Chi-Square test. Conditional logistic regression and unconditional logistic regression were used to analyze the risk factors for SSNHL. The collinearity of all continuous variables was examined before performing the logistic regression using the variance inflation factor (<10). Spearman correlation analysis was used to explore the relationship between lipid parameters, hearing loss and hearing gain.

## Results

### Baseline data for control and SSNHL participants

The SSNHL group exhibited significantly higher prevalence rate of hypertension and higher levels of body mass index (BMI), TC, LDL-C, nonHDL-C, APOB, APOE, ATH index, AI, CRI-I, CRI-II, and LCI compared to the control group, with statistical significance (Table 1).

**Table 1:** Baseline data for control and SSNHL participants.

Variables	Control (n = 226)	SSNHL (n = 452)	*p*-value
Baseline characteristics			
Age (years)^a^	46.00 (36.00–57.00)	48.00 (35.00–59.75)	0.432
Gender (male, %)^b^	95 (42.04%)	193 (42.70%)	0.869
BMI^a^	22.49 (20.79–24.78)	23.44 (21.22–25.39)	0.038
Hypertension (%)^b^	22 (9.73%)	84 (18.58%)	0.003
Laboratory variables			
TC (mmoL/L)^a^	4.72 (4.00–5.28)	5.17 (4.54–5.86)	<0.001
TG (mmoL/L)^a^	0.91 (0.64–1.29)	0.82 (0.58–1.25)	0.248
HDL-C (mmoL/L)^a^	1.38 (1.17–1.56)	1.41 (1.19–1.60)	0.244
LDL-C (mmoL/L)^a^	2.63 (2.18–3.13)	3.01 (2.53–3.60)	<0.001
nonHDL-C (mmoL/L)^a^	3.29 (2.71–3.83)	3.77 (3.16–4.43)	<0.001
Lipoprotein a (mg/dL)^a^	12.85 (6.78–27.40)	11.54 (5.10–26.98)	0.434
APOA1 (mg/dL)^a^	129.00 (114.00–146.00)	131.00 (116.00–148.00)	0.368
APOB (mg/dL)^a^	76.00 (64.75–89.00)	91.00 (76.00–107.75)	<0.001
APOE (mg/dL)^a^	3.95 (3.20–4.75)	4.58 (3.96–5.44)	<0.001
AIP^a^	−0.17 (−0.36 to −0.02)	−0.23 (−0.40 to −0.02)	0.114
ATH index^a^	1.43 (1.04–1.92)	1.87 (1.18–2.86)	<0.001
AI^a^	2.40 (1.94–2.96)	2.68 (2.08–3.41)	<0.001
CRI-I^a^	3.40 (2.94–3.96)	3.68 (3.08–4.41)	<0.001
CRI-II^a^	1.96 (1.56–2.43)	2.20 (1.71–2.75)	<0.001
LCI^a^	8.34 (5.12–13.97)	8.91 (5.45–17.14)	0.023
cLTI^a^	40.75 (14.59–90.00)	36.65 (12.42–85.38)	0.214
cLPI^a^	−45.79 (−65.74 to −5.87)	−39.09 (−63.10–17.57)	0.141

a Values were given as median with its interquartile range (25–75th) in parentheses.

b Values were given as the number of cases and the percentage in parentheses.

### Regression analysis among control and SSNHL groups

**Fig. 1:**
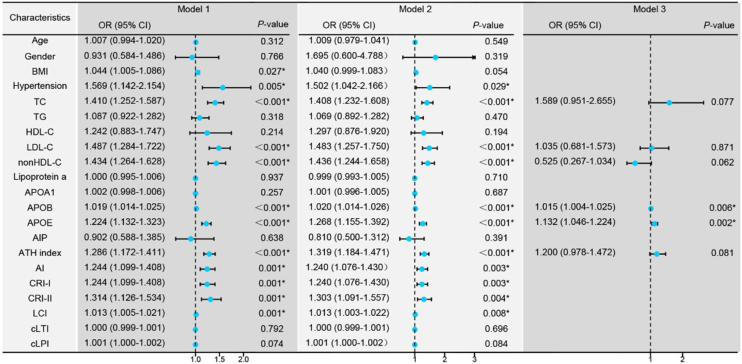
Conditional logistic regression models comparing the control group with the Sudden Sensorineural Hearing Loss (SSNHL) group. Model 1: Univariate logistic regression analysis comparing the control and SSNHL groups. Model 2: Logistic regression analysis adjusted for the confounding factors of age, gender, Body Mass iIndex (BMI), and hypertension. Model 3: Multivariate logistic regression analysis incorporating variables with a *p*-value < 0.001 from the initial analyses. * *p-*value < 0.05 denotes statistical significance.

Conditional logistic regression analysis revealed that BMI, hypertension, traditional lipid parameters (TC and LDL-C), and non-traditional lipid parameters (nonHDL-C, APOB, APOE, ATH index, AI, CRI-I, CRI-II, and LCI) were risk factors for the onset of SSNHL (Fig. 1, Model 1). Even after adjusting for confounding factors including age, gender, BMI, and hypertension (Fig. 1, Model 2), the aforementioned lipid parameters remained significant. Due to strong collinearity among lipid parameters, we only included indicators with *p*-value < 0.001 to construct a multiple logistic regression model and determined that APOB and APOE as independent risk factor for the onset of SSNHL (Fig. 1, Model 3).

### Characteristics of SSNHL patients by hearing loss < 50 dB and ≥ 50 dB

The group with hearing loss ≥ 50 dB had higher age, higher prevalence of hypertension, higher proportion of AF-SSNHL, higher levels of TC, TG, LDL-C, nonHDL-C, AIP, AI, ATH index, CRI-I, CRI-II, and LCI, but lower incidence of ear fullness (Table 2).

**Table 2:** Characteristics of SSNHL patients by hearing loss and prognosis.

Variables	Hearing loss			Therapeutic effect		
	< 50 dB (n = 190)	≥ 50 dB (n = 262)	*P*-value	Effective (n = 206)	Ineffective (n = 246)	*P*-value
**Baseline characteristics**						
Age (years)^a^	44.00 (33.00–58.00)	51.00 (35.75–61.25)	0.012	43.00 (33.00–57.00)	52.00 (38.00–62.25)	0.001
Gender (male, %)^b^	78 (41.05%)	115 (43.89%)	0.547	85 (41.26%)	108 (43.90%)	0.572
BMI^a^	22.88 (20.85–25.19)	23.51 (21.43–25.88)	0.060	23.44 (21.23–25.26)	23.15 (21.15–25.41)	0.965
Hypertension (%)^b^	24 (12.63%)	60 (22.90%)	0.006	34 (16.51%)	50 (20.33%)	0.298
Tinnitus (%)^b^	151 (79.47%)	195 (73.31%)	0.211	159 (77.19%)	187 (76.02%)	0.770
Vertigo (%)^b^	50 (26.32%)	78 (29.77%)	0.421	52 (25.24%)	76 (30.89%)	0.184
Ear fullness (%)^b^	107 (56.32%)	101 (38.55%)	<0.001	102 (49.52%)	106 (43.09%)	0.172
Type (AF-SSNHL, %)^b^	76 (40.00%)	239 (91.22%)	<0.001	145 (70.39%)	171 (69.51%)	0.840
Time to treatment (days)^a^	5.00 (2.00–10.00)	4.00 (2.00–7.00)	0.079	4.00 (2.00–7.00)	5.00 (3.00–13.50)	0.001
Hearing loss (dBHL)^a^	33.00 (25.00–40.00)	80.50 (62.75–96.00)	<0.001	54.09 (34.50–79.00)	57.00 (33.75–88.25)	0.390
Hearing gain (dB)^a^	5.00 (−1.00–12.00)	11.50 (1.00–27.00)	<0.001	18.52 (13.00–31.25)	0.67 (−2.00–4.00)	<0.001
**Laboratory variables**						
TC (mmoL/L)^a^	5.03 (4.51–5.67)	5.30 (4.56–6.07)	0.048	5.27 (4.52–5.82)	5.09 (4.55–6.00)	0.658
TG (mmoL/L)^a^	0.73 (0.56–1.08)	0.90 (0.60–1.39)	0.013	0.79 (0.57–1.25)	0.84 (0.60–1.25)	0.701
HDL-C (mmoL/L)^a^	1.44 (1.21–1.64)	1.37 (1.17–1.58)	0.031	1.41 (1.20–1.62)	1.41 (1.17–1.58)	0.371
LDL-C (mmoL/L)^a^	2.95 (2.52–3.38)	3.10 (2.54–3.75)	0.009	3.01 (2.54–3.59)	3.02 (2.53–3.63)	0.933
nonHDL-C (mmoL/L)^a^	3.62 (3.12–4.15)	3.87 (3.19–4.59)	0.004	3.75 (3.18–4.45)	3.77 (3.12–4.42)	0.91
Lipoprotein a (mg/dL)^a^	11.06 (5.30–27.36)	12.58 (5.06–25.15)	0.744	12.41 (5.28–27.10)	11.00 (4.98–26.95)	0.575
APOA1 (mg/dL)^a^	134.0 (118.0–150.0)	130.0 (114.0–145.0)	0.004	130.0 (117.8–146.3)	133.0 (113.0–149.3)	0.893
APOB (mg/dL)^a^	90.00 (76.00–103.25)	92.00 (76.00–110.00)	0.205	90.00 (77.00–108.00)	92.50 (76.00–107.00)	0.866
APOE (mg/dL)^a^	4.53 (4.01–5.25)	4.61 (3.84–5.54)	0.598	4.63 (4.02–5.57)	4.54 (3.94–5.31)	0.401
AIP^a^	−0.30 (−0.42−0.10)	−0.19 (−0.38‒0.04)	0.001	−0.23 (−0.41−0.04)	−0.24 (−0.49−0.01)	0.587
ATH index^a^	1.65 (1.01–2.59)	2.05 (1.20–3.09)	0.004	1.76 (1.13–2.86)	1.93 (1.14–2.93)	0.509
AI^a^	2.55 (2.00–3.14)	2.83 (2.18–3.58)	0.001	2.60 (2.11–3.31)	2.78 (2.05–3.45)	0.478
CRI-I^a^	3.55 (3.00–4.14)	3.83 (3.18–4.58)	0.001	3.60 (3.11–4.31)	3.78 (3.05–4.45)	0.478
CRI-II^a^	2.08 (1.60–2.54)	2.32 (1.80–2.84)	0.001	2.16 (1.74–2.66)	2.24 (1.70–2.79)	0.488
LCI^a^	8.21 (5.23–13.07)	10.05 (5.89–21.36)	0.001	8.44 (5.36–16.72)	9.21 (5.58–17.37)	0.536
cLTI^a^	34.11 (11.34–88.43)	39.40 (13.36–85.29)	0.36	36.04 (13.15–76.39)	36.79 (11.87–89.56)	0.869
cLPI^a^	−39.35 (−63.37‒17.99)	−38.32 (−62.82‒17.70)	0.81	−40.10 (−63.97‒18.46)	−38.56 (−62.22‒17.57)	0.813

a Values were given as median with its interquartile range (25–75th) in parentheses.

b Values were given as the number of cases and the percentage in parentheses.

### Regression analysis for the different degrees hearing loss

**Fig. 2:**
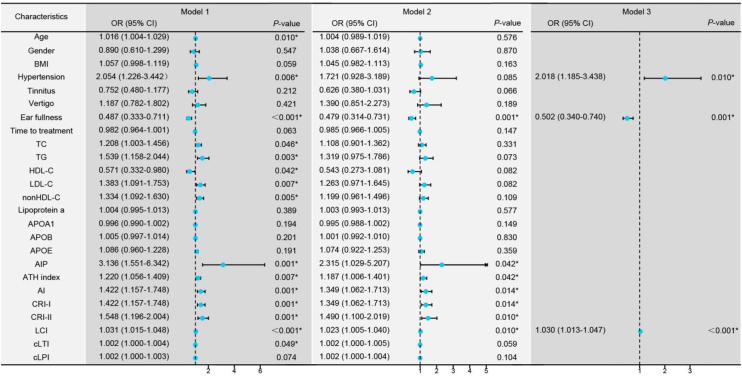
Regression models comparing groups with hearing loss < 50 dB versus ≥ 50 dB. Model 1: Univariate logistic regression analysis for the different degrees of hearing loss. Model 2: Multivariate logistic regression analysis controlling for confounding factors including age, gender, Body Mass Index (BMI), hypertension, tinnitus, vertigo, ear fullness, and time to treatment initiation (days). Model 3: Forward logistic regression analysis including variables with a *p*-value < 0.01 from Model 1. *p-value < 0.05 is considered statistically significant.

Univariate logistic regression revealed that age, hypertension, ear fullness, TC, TG, HDL-C, LDL-C, nonHDL-C, AIP, ATH index, AI, CRI-I, CRI-II, LCI, and cLTI were influencing factors for the degree of hearing loss (Fig. 2, Model 1). These variables were adjusted for confounding factors including age, gender, BMI, hypertension, tinnitus, vertigo, ear fullness and time to treatment (Fig. 2, Model 2), and found ear fullness, comprehensive lipid parameters (AIP, ATH index, AI, CRI-I, CRI-II, and LCI) remained significant. Due to strong collinearity, forward logistic regression was adopted to analyze the variables with *p*-value < 0.05 in univariate logistic regression and found that hypertension, ear fullness, and LCI were ultimately identified as the independent determinants (Fig. 2, Model 3).

### Regression analysis of hearing loss among PF-SSNHL and AF-SSNHL groups

**Fig. 3:**
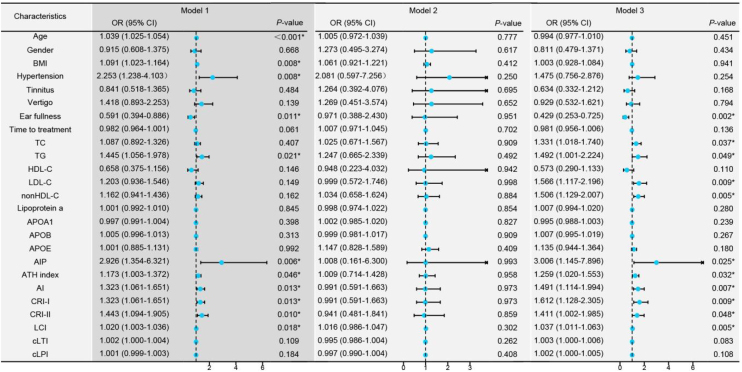
Univariate logistic regression models among the different types of Sudden Sensorineural Hearing Loss (SSNHL). Model 1: Univariate logistic regression analysis comparing Partial Frequency descending SSNHL (PF-SSNHL) with All Frequency descending SSNHL (AF-SSNHL). Model 2: Univariate logistic regression analysis for the different degrees of hearing loss in PF-SSNHL. Model 3: Univariate logistic regression analysis for the different degrees of hearing loss in AF-SSNHL. * *p*-value < 0.05.

The higher the age, BMI, prevalence of hypertension, TG, AIP, AI, ATH index, CRI-I, CRI-II, and LCI, the higher the incidence of AF-SSNHL. However, ear fullness indicated the occurrence of PF-SSNHL (Fig. 3, Model 1). Moreover, blood lipids mainly affected the degree of hearing loss in patients with AF-SSNHL (Fig. 3, Model 3).

### Characteristics of SSNHL patients by prognosis

**Fig. 4:**
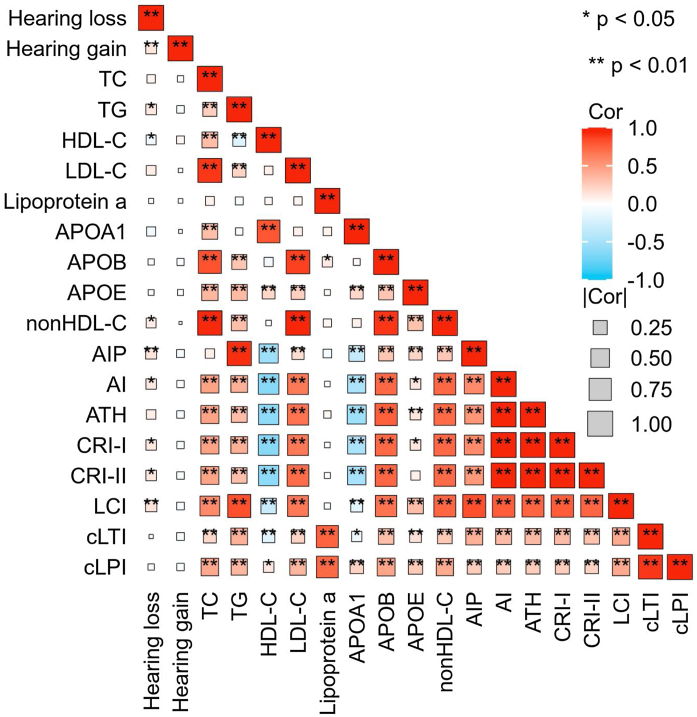
Correlation Heatmap. This heatmap depicts the strength of the correlations between lipid expression levels and degrees of hearing loss and gain. The size of each box indicates the magnitude of the correlation, while the color hue reflects the direction of the association. Deeper shades of red indicate a stronger positive correlation, whereas deeper shades of blue suggest a stronger negative correlation.

SSNHL patients with hearing improvement <10 dB generally had a higher age and a longer time from hearing loss to seeking medical attention. However, there was no difference in blood lipid parameters between the effective and ineffective groups (Table 2). Spearman correlation analysis revealed a linear correlation between TG (*r* = 0.117, *p* = 0.018), HDL-C (*r* = −0.095, *p* = 0.045), AIP (*r* = 0.135, *p* = 0.004), nonHDL-C (*r* = 0.093, *p* = 0.0.047), AI (*r* = 0.111, *p* = 0.018), CRI-I (*r* = 0.111, *p* = 0.018), CRI-II (*r* = 0.113, *p* = 0.016), LCI (*r* = 0.145, *p* = 0.002) and the level of hearing loss. However, there was no linear relationship observed between hearing gain and any lipid parameters (Fig. 4).

### Regression analysis among the effective and ineffective groups

**Fig. 5:**
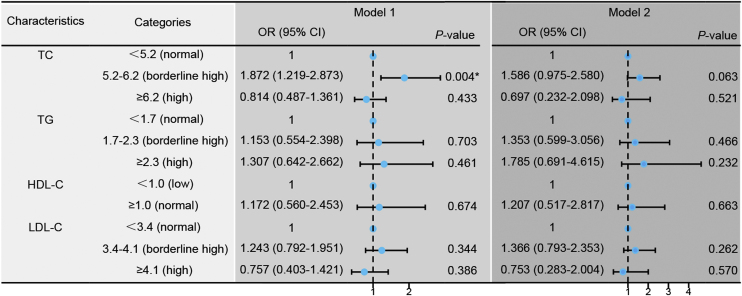
Logistic regression models comparing treatment efficacy between the effective and ineffective groups. Model 1: Univariate logistic regression analysis to compare the two groups based on treatment effectiveness. Model 2: Adjusted logistic regression analysis for confounding factors including age, gender, Body Mass Index (BMI), hypertension, tinnitus, vertigo, ear fullness, time to initiation of treatment (days), type of intervention, and degree of Hearing Loss (dBHL). *p < 0.05 indicates statistical significance.

According to the Chinese guideline for the management of dyslipidemia in adults, TC, TG, LDL-C, and HDL-C were divided into normal, borderline high, and high categories.^12^ Then it was found that when TC was in the range of borderline high, the therapeutic effect was the best. The response rate decreased when TC was in the normal range or in a high-risk state (Fig. 5, Model 1). However, the statistical significance disappeared upon adjusting for confounding factors including age, gender, BMI, anti-hypertensive therapy, lipid-lowering therapy, tinnitus, vertigo, ear fullness, time to treatment, type, hearing loss, suggesting the existence of some factors that exert a more pronounced impact on the hearing improvement of SSNHL (Fig. 5, Model 2).

## Discussion

Dyslipidemia can hinder microcirculation within the inner ear, leading to the disappearance of the endocochlear potential.^13^ Moreover, lipid metabolism and distribution play critical roles in the cochlea's energy supply and signal transduction.^14^, ^15^ It is speculated that the increase of blood lipids may be one of the causes of hearing loss by blocking microcirculation and affecting lipid distribution in the inner ear. This study found that lipid parameters were significantly associated with the occurrence of SSNHL and the degree of hearing loss. APOB, APOE, and LCI, were identified as independent risk factors. Certain lipids showed a positive linear correlation with hearing loss, but not hearing gain. When TC was in borderline high range, the treatment effect was the best.

A recent meta-analysis found that patients presenting with SSNHL had a significantly higher risk of concomitant hypertension and higher TC compared to matched controls, but not LDL-C, HDL-C, and TG.^16^ There was also evidence supporting that TC, LDL-C, APOA1, APOB, nonHDL-C, AIP, ATH index, CRI-I, CRI-II, and fatty acids were risk factors for SSNHL.^3^, ^16^, ^17^, ^18^, ^19^, ^20^ This study further corroborated the heightened risk of concurrent hypertension and dyslipidemia in SSNHL patients, particularly in those with hearing loss of ≥50 dB and AF-SSNHL.

In the multivariate logistic regression model, non-traditional lipid parameters demonstrated a stronger explanatory power for the outcomes observed. Atherogenic lipoproteins (APOB-containing lipoproteins), but not by their cholesterol content or the type of lipoproteins, are the most important attributes for determining the risk of Atherosclerotic Cardiovascular Disease (ASCVD), and APOB is proposed as a superior proxy to LDL-cholesterol.^21^ The APOE ε4 allele may trigger pro-inflammatory pathways in pericytes, thus raising susceptibility to blood-brain barrier damage, reducing pericyte coverage, and diminishing microvascular length.^22^ Unfortunately, our study did not determine specific APOE alleles. Comprehensive lipid indices reflect the balance between proatherogenic and antiatherogenic lipoprotein particles and provide a better assessment of overall lipid metabolism status and the risk of ASCVD compared to single traditional blood lipid parameters. Although study had reported LCI as a risk factor for ASCVD, the effect of LCI on SSNHL remains unexplored.^9^

The impact of blood lipids on hearing loss was primarily evident in AF-SSNHL, as opposed to PF-SSNHL. AF-SSNHL pathology is predominantly tied to blood supply disruptions and vascular embolism, while PF-SSNHL relates to endolymphatic hydrops or external hair cell damage.^10^ Furthermore, age, hypertension and ear fullness were also important determinants in affecting the degree of hearing loss and type of SSNHL. Ear fullness mainly occured in low-frequency SSNHL with a tendency to develop endolymphatic hydrops, so patients with ear fullness had a milder degree of hearing loss.^10^, ^23^

Numerous studies have attempted to clarify the role of serum lipids in determining the prognosis of SSNHL, yet their findings diverge. While Quaranta N, et al.^24^ suggested that higher TC levels correlated with poorer hearing recovery rates. Li XQ, et al.^25^ challenged this view, placing greater emphasis on APOB as a critical prognostic factor. Interestingly, our results align with a study that observed an optimal treatment outcome with slight elevations in TC levels, rather than when TC levels were above 6.2 mmol/L or within a normal range.^26^ Cochlear is highly susceptible to changes in blood flow. In symptomatic men with significant coronary atherosclerosis and normal to moderately elevated serum cholesterol, less progression of coronary atherosclerosis and fewer new cardiovascular events were observed in the group of patients treated with lipid-lowering therapy.^27^ Thus, it's a reasonable guess that the improvement in microcirculation can lead to significant hearing recovery when TC was borderline high. Treatment efficacy diminished in cases of complete microvascular obstruction or non-microcirculatory etiologies of hearing loss. One might surmise that the discrepancies noted in previous studies could be due to treatment protocols, patient cohorts, prognostic evaluation criteria, and statistical methodologies, which are areas current researches seek to standardize. Despite data variability, research generally indicates a correlation between lipid levels and hearing recovery.^24^, ^25^, ^26^ Previous research emphasized hyperlipidemia's detrimental effects on the inner ear, with lipid reduction alleviating SSNHL.^28^ Thus, for SSNHL patients with marginally elevated TC, focus should be on enhancing microcirculation. For SSNHL patients with marked hypercholesterolemia, consideration should be given to more prompt and efficacious lipid-lowering interventions.

Although this study attempts to control for confounding factors to minimize the impact of specific diseases on lipid metabolism, diet, sleep, and lifestyle habits may still affect lipid levels and may be difficult to control for in the study. Moreover, excluding participants with diseases that interfere with lipid metabolism could result in a lack of representativeness for the broader real-world population. Our investigation is retrospective, and a causal relationship between lipid levels and outcomes is not established. The effect of TC on prognosis is a reasonable inference based on initial results and warrants further validation through more rigorous clinical trials.

## Conclusion

Patients with SSNHL exhibited markedly dysregulated lipid metabolism. Elevated serum lipid levels may be a causative factor in auditory impairment and can influence the extent of hearing loss. Promptly improving cochlear microcirculation may benefit patients with borderline elevated TC.

## Authors’ contributions

DY Xie and YM Feng designed and supervised the project. XM Liu and JN Huang were responsible for the collection of blood samples and data. XY Chen and Z Zheng contributed to the statistical analysis and interpretation of data and wrote the first draft of the paper. All authors contributed to the revision of the article and approved the final published version to be published.

## Funding

This study was supported by Shanghai Shenkang Hospital Development Center (SHDC2022CRD010); The National Natural Science Foundation of China (82171139); Shanghai Municipal Health Commission (2023ZZ02008).

## Ethics committee

Sixth People's Hospital Affiliated to Shanghai Jiaotong University [2018-KY-036(K), 2018.07.24].

## Conflicts of interest

The authors declare no conflicts of interest.
